# Intraspecific DNA Barcoding and Variation Analysis for Citri Reticulatae Pericarpium of *Citrus reticulata* “Chachi”

**DOI:** 10.1155/2021/2609935

**Published:** 2021-12-09

**Authors:** Mengshi Liu, Kanghui Wang, Baizhong Chen, Yi Cai, Chuwen Li, Wanling Yang, Minyan Wei, Guodong Zheng

**Affiliations:** ^1^Key Laboratory of Molecular Target & Clinical Pharmacology and the State Key Laboratory of Respiratory Disease & The Fifth Affiliated Hospital, School of Pharmaceutical Sciences, Guangzhou Medical University, Guangzhou 511436, China; ^2^Guangdong Xinbaotang Biological Technology Co., Ltd, Guangdong, Jiangmen 529000, China

## Abstract

Citri Reticulatae Pericarpium, the desiccative mature peel of *Citrus reticulata* Blanco or its cultivated varieties, is a national geographical indicated product that has the concomitant function of both medicine and foodstuff. The primary source of Citri Reticulatae Pericarpium is *Citrus reticulata* “Chachi,” called “Guang chenpi,” while it differs in variety, propagation, grafting rootstock, and tree age, and the hereditary stability of its biological information between intraspecific plants is worthy of our attention. Homologous analysis result of 4 DNA barcodings in the ribosome or the chloroplast showed that the homology of them (ITS2, rbcl, matK, and psbA-trnH) of 22 samples was 100.00%, 99.97%, 99.99%, and 99.81%, respectively, which indicated that 4 DNA barcodes maintained a high degree of genetic stability in *Citrus reticulata* “Chachi.” Also, ITS2 was considered to identify *Citrus reticulata* “Chachi” from other varieties because it presented not only low variability within a certain taxon but also a high level of interspecies variability. Simultaneously, variant site detection of *Citrus reticulata* “Chachi” was analyzed by comparing with the reference *Citrus reticulata* genome, and 2652697 SNP sites and 533906 InDel sites were detected from whole-genome resequencing data of 22 samples, providing the data resources and theoretical foundation for the future study about the relevant molecular makers of “Guang chenpi.”

## 1. Introduction

Citri Reticulatae Pericarpium (CRP), a traditional Chinese medicine that has the concomitant function of both medicine and foodstuff, is the desiccated mature peel of *Citrus reticulata* Blanco or its cultivated varieties. Thereinto, *Citrus reticulata* “Chachi,” the main cultivated variety of *Citrus reticulata* Blanco, is the primary source of genuine Chinese medicinal materials “Guang Chenpi” [1]. As a national product of geographical indication, “Guang Chenpi” is widely used in clinical applications and by-product processing because of its better quality in CRP [2–4], and most studies have shown that its pharmacological activities include antiasthmatic effects, antineuroinflammatory activity, antioxidant ability, and anticancer activity [5–7].

Up to now, a large number of studies have focused on the chemical compounds in CRP by morphological identification, microscopic identification, TLC, UV, HPLC, GC-MS, and LC-MS [8–10], while these method do not distinguish well among different cultivars or among different varieties of *Citrus reticulata* “Chachi.” As an emerging method of identification of food and natural medicinal materials, molecular marker (DNA barcoding, SNP, and InDel) has considerable untapped potential in the quality control and origin identification of food and medicinal materials. DNA barcoding, an important tool for ecological research, has been widely used in species identification [11–14]. A number of studies have shown that plant DNA core barcodes are used internationally in the fields of species discovery, taxonomy, flora, and ecology [15–17]. Nevertheless, molecular marker about different cultivars of CRP or different varieties of *Citrus reticulata* “Chachi” was less studied. Previous studies have reported that the ITS2 region was selected for discrimination of the four CRP cultivars; however, this study did not take the intraspecific variation of *Citrus reticulata* “Chachi” into consideration [18]. Between different plants of *Citrus reticulata* “Chachi,” they showed some difference in the tree age and the variety including big-leaf species small-leaf species. Besides, propagations of *Citrus reticulata* “Chachi” include layerage on its maternal plant or graftage on different rootstocks such as *Citrus limonia* Osbeck, *Citrus reticulata* Blanco, and *Poncirus trifoliata* (L.) Raf.

Herein, 4 DNA barcodings including ITS2, rbcl, matK, and psbA-trnH were chosen for biological evolutionary information analysis about *Citrus reticulata* “Chachi” of different propagation methods, different tree ages, different varieties, and different rootstocks. Among them, ITS2 is a segment of DNA in the ribosome [19], and rbcl, matK, and psbA-trnH are DNA fragments in the chloroplast.

Except for a study on genetic stability of 4 barcodes, genetic diversity analysis of *Citrus reticulata* “Chachi” was carried out thought whole-genome resequencing technology with DNBSEQ-T7, compared with the reference published genomic data of *Citrus reticulata* from the NCBI (GenBank accession number ASM325862v1) [20], further excavating single-nucleotide polymorphism (SNP) sites and insertion-deletion (InDel) sites from whole-genome resequencing data of 22 *Citrus reticulata* “Chachi” samples.

The objective of this work was to research the hereditary stability of 4 DNA barcodings (ITS2, rbcl, matK, and psbA-trnH) in different *Citrus reticulata* “Chachi” plants, which can provide screening indicator of DNA barcoding to distinguish *Citrus reticulata* “Chachi” and other varieties of CRP. Also, variant type detection based on whole-genome resequencing data provides more potential molecular markers to distinguish *Citrus reticulata* “Chachi” between intraspecific plants or other cultivars, laying a foundation for the further development of “Guang chenpi.”

## 2. Materials and Methods

### 2.1. Biological Materials

Twenty-two batches of biological materials were collected from the Germplasm Source and Seedling Breeding Center of “Guang chenpi” (Table 1). 22 *Citrus reticulata* “Chachi” samples were different in variety, plant propagation, rootstock, and tree age.

### 2.2. DNA Extraction

Genomic DNA was extracted using the plant DNA extraction kit (TSP101-200) of Tsingke. The quality of the extracted genomic DNA was checked by 1% agarose gel electrophoresis with DL2000 DNA marker, and the concentration of them was carried out through the NanoDrop 1000 (Thermo Fisher Scientific, Waltham Massachusetts, US).

### 2.3. PCR and Sequencing of DNA Barcodings

Genomic DNA was diluted to 15 ng·*μ*l^−1^ and then was amplified by performing polymerase chain reaction (PCR) using 4 pair of universal primers of DNA barcodings listed in Table 2 [18]. PCR was performed under the following conditions: initial denaturation at 98°C for 2 min, followed by 30 cycles with 98°C denaturation for 10 s, annealing at the melting temperatures (TM) listed in Table 2 for 10 s, and extension at 72°C for 10 s. The final extension step was performed for 5 min at 72°C. Next, an aliquot of the amplification product was resolved on 1% agarose gel electrophoresis documented with a gel documentation system and further analyzed by sequencing.

### 2.4. DNA Library Construction and Illumina Sequencing

Genomic DNA will be randomly interrupted, the end will be repaired, “A” will be added, and the unique connector of DNBSEQ-T7 sequencer will be added. Then, DNA libraries will be constructed by PCR enrichment. Finally, the DNA library was denatured, cycled, and digested to obtain single-stranded circular DNA. Single-stranded circular DNA was amplified by rolling circle amplification (RCA), further producing DNA nanoball (DNB). Illumina sequencing was performed on DNBSEQ-T7 sequencer after the DNA libraries were qualified.

### 2.5. Whole-Genome Resequencing Data Quality and Filtering

To exclude bias from low-quality reads that arise from the process of base-calling or adapter contamination, the quality of the raw data obtained by whole-genome resequencing was evaluated until the value of *Q*_30_ was over than 85%. The clean reads were used for subsequent bioinformatics analysis. For further analysis, we downloaded previously published genomic data of *Citrus reticulata* from the NCBI (GenBank accession number ASM325862v1). We mapped high-quality data per individual to the reference *Citrus reticulata* genome using Burrows–Wheeler Aligner (BWA) software [21], then the sequencing read depth and genomic coverage of each sample were counted, and the variation was detected.

### 2.6. SNP and InDel Calling

SNPs and InDels can be called by mapping the unitigs against a reference genome. The main calling procedures are as follows: (1) for the results of BWA comparison, Mark Duplicate tool of Picard software is used to remove the duplication and shield the influence of PCR-duplication; (2) the Genome Analysis Toolkit (GATK) software [22] is used to perform InDels realignment, with local realignment of the sites near the alignment result with insertion-miss alignment and correction of alignment errors due to insertion-miss alignment; (3) GATK software was used for base recalibration to calibrate the base masses; (4) variant calling of SNPs and InDels was performed by GATK software; and (5) SNPs and InDels with any of the following features were filtered: two SNPs within 5 bp; SNPs within 5 bp near InDel; and two InDels within 10 bp [23].

## 3. Results and Discussion

### 3.1. Quality and Concentration of the Extracted DNA

In this work, Genomic DNA was extracted from tender leaves of 22 *Citrus reticulata* “Chachi” samples by using the plant DNA extraction kit, and OD value (A260/280) and DNA concentration are shown in Table 3. The results showed that the concentration of DNA could be used in subsequent experiments.

### 3.2. Sequence Features and Homologous Analysis of DNA Barcodings

**Figure 1:**
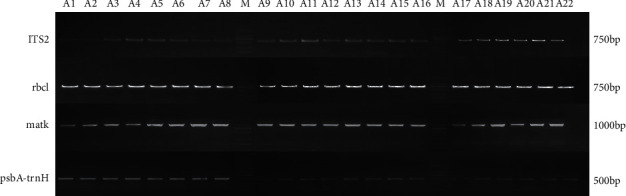
Agarose gel electrophoresis of ITS2, rbcl, matK, and psbA-trnH.

According to the agarose gel electrophoresis result of PCR amplification products (Figure 1), ITS2, rbcl, matK, and psbA-trnH produced amplification bands of approximately 750 bp, 750 bp, 1000 bp, and 500 bp, respectively. The electrophoresis bands of each sample were uniform, bright, and nonspecific heterozygous, indicating that the success rate of sequence amplification was 100%, which could be further analyzed by sequencing.

Four barcodes (ITS2, rbcl, matK, and psbA-trnH) were analyzed by DNAMAN software for the length and base composition of each sequence fragment and further identified by BLAST in Genebank. The success rates of PCR amplification and sequencing of 3 DNA barcodings (ITS2, rbcl, and matK) of 22 samples were 100%. However, due to the large number of fragments missing in the sequencing of two samples (A16 and A21) of the psbA-trnH barcode, 20 psbA-trnH sequences were actually obtained in the experiment. PCR success rate, barcoding length, GC content, variable site, and BLAST rate of 4 DNA barcodings are listed in Table 4. The aligned partial sequences had lengths of 232 bp, 680∼682 bp, 1059 bp, and 537∼538 bp for ITS2, rbcl, matK, and psbA-trnH, respectively. Among them, the ITS2 barcode had the advantages of shorter sequence length and higher GC content (71.60%), followed by the psbA-trnH barcode having shorter sequence length.

Moreover, homologous analysis of 4 DNA barcodings about 22 batches of *Citrus reticulata* “Chachi” samples was carried out by DNAMAN software. The homologous analysis result showed that the homology of ITS2 of 22 samples was 100.00%, which indicated that ITS2 maintained a high degree of genetic stability in *Citrus reticulata* “Chachi” of different propagation methods, different tree ages, different varieties, and different rootstocks. Also, the homology of rbcl, matK, and psbA-trnH of 22 samples was 99.97%, 99.99%, and 99.81%, respectively.

In addition, among the 22 batches of CRP samples, 2 SNP sites were identified in the matK barcode and 1 Indel site was identified in the psbA-trnH barcode. The results showed that there were still some variations within the species of *Citrus reticulata* “Chachi,” making us realize molecular breeding of *Citrus reticulata* “Chachi” and the distinction of *Citrus reticulata* “Chachi” and related species need more valuable molecular markers. Therefore, whole-genome resequencing was also performed on 22 *Citrus reticulata* “Chachi” samples of different propagation methods, different tree ages, different varieties, and different rootstocks, which provided more scientific basis for molecular breeding.

Previous studies have reported that the ITS2 region was selected for discrimination of the four CRP cultivars including *Citrus reticulata* “Chachi,” *Citrus reticulata* “Dahongpao,” *Citrus reticulata* “Unshiu,” and *Citrus reticulata* “Tangerina,” while ITS, trnH-psbA, and rbcL could not distinguish these CRP samples [18]. Different from the existing studies, this work focuses on the hereditary stability of 4 barcodes including ITS2, rbcl, matK, and psbA-trnH in *Citrus reticulata* “Chachi” with different varieties, propagation methods, grafting rootstocks, and tree ages. Because of DNA degradation in moderately or highly processed products with time, PCR amplification of standard-length (around 650 bp) barcodings is a huge challenge [24]. Combined with existing research and the result in this work, ITS2 was considered to be a useful DNA barcoding to distinguish *Citrus reticulata* “Chachi” from other varieties, which presented not only low variability within a certain taxa but also a high level of interspecies variability. Also, this work indicated that matK was not considered because of its long length and variable sites within taxa, while rbcl and psbA-trnH had the potential to distinguish *Citrus reticulata* “Chachi” from other varieties. Actually, combining DNA barcodes in the ribosome and in the chloroplast makes it more convincing in species identification of plants [25, 26].

### 3.3. Quality Analysis of Whole-Genome Resequencing Data

A total of 22 *Citrus reticulata* “Chachi” sample genomes were sequenced, which generated 158 Gb raw data. Base coverage depth distribution curve and coverage distribution curve indicated that the coverage depth of the bases on the genome was evenly distributed. The statistical results of insert fragment distribution with a single peak show that insert fragment distribution fits the normal distribution and the construction of DNA libraries was reliable. The chromosome coverage depth map showed that the genome was evenly covered, indicating good randomness of sequencing.

Summary of clean sequencing data results about 22 *Citrus reticulata* “Chachi” samples is given in Table 5. The size of reference genome *Citrus reticulata* is 344.27 Mb (assembly level: scaffold). In this work, the average coverage depth was 16X, and the value of *Q*_30_ reached 88.94%. The average mapped ratio and genome coverage of all the samples were 98.97% and 93.55%, respectively. The average GC content of *Citrus reticulata* “Chachi” was 38.68% in line with reference genome *Citrus reticulata*.

### 3.4. SNP and InDel Calling of *Citrus reticulata* “Chachi”

In this study, the variant site detection of *Citrus reticulata* “Chachi” for the national geographical indicated product CRP was firstly analyzed by SNP and InDel calling from whole-genome resequencing data (Table 6). Except for the high genetic stability of 4 barcodes (ITS2, rbcl, matK, and psbA-trnH), 22 *Citrus reticulata* “Chachi” samples showed its genetic diversity between different propagation methods, different tree ages, different varieties, and different rootstocks as well.

A total of 2652697 SNP sites were excavated between 22 *Citrus reticulata* “Chachi” samples, among which 1741507 SNP sites were transition (T_i_), 902182 SNP sites were transversion (T_v_), and 9008 SNP sites were transition or transversion. These SNP sites were with a T_i_/T_v_ ratio of 1.93, which is in line with general rules of base mutation in natural organisms [27]. In the course of evolution about *Citrus reticulata* “Chachi,” transition happens much more frequently than transversion, which means that evolution of *Citrus reticulata* “Chachi” tends to accept the substitution between purines and purines or the substitution between pyrimidines and purines, the substitution between purines and pyrimidines causes bad things to happen, and that substitution has mostly been eliminated by evolution.

In addition, InDel sites, as codominant molecular markers, are widely distributed in the genome with high density, which are suitable for genome-wide molecular marker exploration. A total of 533906 InDel sites were detected between 22 *Citrus reticulata* “Chachi” samples, among which 275380 InDel sites were insertion, 241768 InDel sites were deletion, and 9008 InDel sites were insertion or deletion. Unlike SNP sites, insertions and deletions of InDel sites are equally likely to occur.

## 4. Conclusions

Overall, our work indicated that 4 DNA barcodes (ITS2, rbcl, matK, and psbA-trnH) maintained a high degree of genetic stability in *Citrus reticulata* “Chachi” of different propagation methods, different tree ages, different varieties, and different rootstocks. Because ITS2 presented not only low variability within a certain taxa but also a high level of interspecies variability, it was considered to be an useful DNA barcoding to identify *Citrus reticulata* “Chachi” from other varieties. Moreover, 2652697 SNP sites and 533906 InDel sites were detected from whole-genome resequencing data of 22 *Citrus reticulata* “Chachi” samples, fully reflecting the genetic diversity of *Citrus reticulata* “Chachi” with different varieties or propagation methods. To excavate more useful molecular markers for distinguishing *Citrus reticulata* “Chachi” between intraspecific plants or other cultivars, DNA barcoding analysis and variant type detection of the *Citrus reticulata* “Chachi” were studied for the first time in this investigation, which laid a special foundation for the biological information analysis of *Citrus reticulata* “Chachi” for the national geographical indicated product Citri Reticulatae Pericarpium.

## Figures and Tables

**Table 1:** Information of *Citrus reticulata* “Chachi” samples.

No.	Sample source	Plant propagation	Variety	Rootstock	Tree age (years)
A1	Tianma Village, Huicheng Town, Xinhui District, Jiangmen City, Guangdong Province	Graftage	Big-leaf	*Citrus limonia* Osbeck	11
A2	Dadong Village, Huicheng Town, Xinhui District, Jiangmen City, Guangdong Province	Graftage	Big-leaf	*Citrus limonia* Osbeck	6
A3	Tianma Village, Huicheng Town, Xinhui District, Jiangmen City, Guangdong Province	Graftage	Big-leaf	*Citrus limonia* Osbeck	8
A4	Tianma Village, Huicheng Town, Xinhui District, Jiangmen City, Guangdong Province	Graftage	Big-leaf	*Citrus limonia* Osbeck	8
A5	Dongjia Village, Huicheng Town, Xinhui District, Jiangmen City, Guangdong Province	Layerage	Big-leaf	—	10
A6	Qunsheng Village, Huicheng Town, Xinhui District, Jiangmen City, Guangdong Province	Layerage	Big-leaf	—	10
A7	Qibao Village, Huicheng Town, Xinhui District, Jiangmen City, Guangdong Province	Graftage	Big-leaf	*Citrus limonia* Osbeck	11
A8	Shenglu Village, Sanjiang Town, Xinhui District, Jiangmen City, Guangdong Province	Graftage	Small-leaf	*Citrus limonia* Osbeck	6
A9	Guangtian Village, Sanjiang Town, Xinhui District, Jiangmen City, Guangdong Province	Graftage	Wild species	*Citrus reticulata* Blanco	5
A10	Shenlu Village, Sanjiang Town, Xinhui District, Jiangmen City, Guangdong Province	Graftage	Big-leaf	*Citrus limonia* Osbeck	5
A11	Shenlu Village, Sanjiang Town, Xinhui District, Jiangmen City, Guangdong Province	Layerage	Big-leaf	—	5
A12	Xinsheng Village, Siqian Town, Xinhui District, Jiangmen City, Guangdong Province	Graftage	Big-leaf	*Citrus limonia* Osbeck	25
A13	Shanyi Village, Siqian Town, Xinhui District, Jiangmen City, Guangdong Province	Graftage	Small-leaf	*Citrus limonia* Osbeck	10
A14	Shanyi Village, Siqian Town, Xinhui District, Jiangmen City, Guangdong Province	Graftage	Small-leaf	*Citrus limonia* Osbeck	10
A15	Shanyi Village, Siqian Town, Xinhui District, Jiangmen City, Guangdong Province	Graftage	Big-leaf	*Poncirus trifoliata* (L.) Raf.	8
A16	Yaqian Village, Shuangshui Town, Xinhui District, Jiangmen City, Guangdong Province	Graftage	Big-leaf	*Citrus limonia* Osbeck	8
A17	Shalu Village, Shuangshui Town, Xinhui District, Jiangmen City, Guangdong Province	Graftage	Big-leaf	*Citrus limonia* Osbeck	5
A18	Guangdong Province seedling breeding farm	Graftage	Big-leaf	*Poncirus trifoliata* (L.) Raf.	10
A19	Guangdong Province seedling breeding farm	Graftage	Big-leaf	*Citrus limonia* Osbeck	6
A20	Wenlong Village, Daze Town, Xinhui District, Jiangmen City, Guangdong Province	Graftage	Big-leaf	*Citrus limonia* Osbeck	10
A21	Yaxi Village, Yamen Town, Xinhui District, Jiangmen City, Guangdong Province	Graftage	Big-leaf	*Citrus limonia* Osbeck	13
A22	Changsha Village, Gujing Town, Xinhui District, Jiangmen City, Guangdong Province	Graftage	Big-leaf	*Citrus limonia* Osbeck	10

**Table 2:** Primer sequence and PCR system of DNA barcodings.

Barcoding	Direction	Primer sequence (5′⟶3′)	PCR system	TM (°C)
ITS2	F	ATGCGATACTTGGTGTGAAT	98°C 2 min; 98°C 10 s, TM °C 10 s, 72°C 10 s; 72°C 5 min	58
	R	GACGCTTCTCCAGACTACAAT		
rbcl	F	ATGTCACCACAAACAGAAAC		58
	R	TCGCATGTACCTGCAGTAGC		
matK	F	AGAGGTATTTGCTGCTGTGGTG		58
	R	GGAAAGAGTAAAGCAAGAACGTGT		
psbA-trnH	F	AGGTATCTGGTTCACTGCTTTAGGT		59
	R	GCCTTGATCCACTTGGCTACAT		

TM, The melting temperature of DNA.

**Table 3:** Quality and concentration of the extracted DNA.

Sample no.	OD value (A260/280)	DNA concentration (ng/*μ*l)	Total DNA (*μ*g)
A1	1.792	178	5.34
A2	1.763	151	4.53
A3	1.671	104	3.12
A4	1.749	240	7.20
A5	1.790	110	3.30
A6	1.115	16	0.47
A7	1.826	153	4.59
A8	1.761	104	3.12
A9	1.707	134	4.02
A10	1.788	186	5.58
A11	1.826	150	4.50
A12	1.882	11	0.32
A13	1.785	125	3.75
A14	1.805	179	5.37
A15	1.816	133	3.99
A16	1.812	166	4.98
A17	1.817	149	4.47
A18	1.656	81	2.42
A19	1.811	112	3.36
A20	1.818	144	4.32
A21	1.740	236	7.08
A22	1.650	120	3.60

**Table 4:** Sequence features of 4 DNA barcodings.

Barcoding	PCR success rate (%)	Barcoding length (bp)	*G* + *C* content (%)	Variable site	BLAST rate (%)
ITS2	100	232	71.60	0	100.00
rbcl	100	680∼682	44.80	0	99.79
matK	100	1059	36.00	2	100.00
psbA-trnH	100	537∼538	32.46	1	99.91

**Table 5:** Summary of sequencing data results about *Citrus reticulata* “Chachi.”

No.	Clean reads	Clean bases	Depth (X)	*Q*_20_ (%)	*Q*_30_ (%)	*G* + *C* content (%)	Read mapping (%)	Genome mapping (%)
A1	49667300	7450095000	17	96.64	89.37	39.04	99.06	94.65
A2	45743350	6861502500	16	95.80	87.28	38.13	98.54	92.53
A3	47316474	7097471100	17	96.33	88.37	37.91	98.98	93.78
A4	41918262	6287739300	15	96.35	88.30	38.14	98.83	93.42
A5	44889792	6733468800	16	96.25	88.36	38.03	98.65	93.06
A6	32910626	4936593900	11	95.86	87.12	38.51	98.86	93.17
A7	45716786	6857517900	16	96.07	88.12	39.22	99.18	94.68
A8	80297744	112044661600	29	96.35	89.09	38.45	99.29	93.83
A9	49858294	7478744100	15	96.71	89.57	40.23	98.81	93.39
A10	43580158	6537023700	15	96.22	88.29	39.44	99.05	94.45
A11	46206262	6930939300	16	96.72	89.52	38.76	98.85	93.56
A12	42274388	6341158200	15	96.74	91.56	38.15	99.02	88.15
A13	53238294	7985744100	18	96.36	88.47	38.62	99.03	93.94
A14	42326498	6348974700	15	96.40	88.89	39.60	99.24	94.72
A15	47956602	7193490300	17	96.52	88.85	38.43	99.04	94.23
A16	47127502	7069125300	16	96.30	88.36	38.03	98.70	92.89
A17	45382574	6807386100	16	96.37	88.52	39.02	99.17	94.85
A18	53972916	8095937400	20	96.33	88.78	37.67	99.15	94.28
A19	48034834	7205225100	17	95.99	87.54	38.21	99.05	93.35
A20	43896212	6584431800	15	96.55	88.99	38.44	98.84	93.40
A21	45485090	6822763500	15	96.42	88.64	39.89	99.10	94.31
A22	43174144	6476121600	14	96.74	89.42	38.98	98.96	93.62

**Table 6:** SNPs and InDels of *Citrus reticulata* “Chachi.”

Variant type	Category	Number	Category/variant type (%)	Total
SNP	Transition	1741507	65.65	2652697
	Transversion	902182	34.01	
	Transition/transversion	9008	0.34	

InDel	Insertion	275380	51.58	533906
	Deletion	241768	45.28	
	Insertion/deletion	16758	3.14	

## Data Availability

The data used to support the findings of this study are included within the article.
